# Genomic Insight into *Escherichia coli* Bloodstream Infections at a Tertiary Hospital in Shanghai: Predominance, Genetic Diversity, and Plasmid-Associated Virulence of CC131 and CC14

**DOI:** 10.3390/antibiotics15100940

**Published:** 2026-09-22

**Authors:** Yibadaiti Tulufu, Qiong Wu, Shuzhen Xiao, Yingzhi Wang, Jianqiang Wang, Lizhong Han, Jin Tang

**Affiliations:** 1 Department of Laboratory Medicine, Ruijin Hospital, Shanghai Jiao Tong University School of Medicine, Shanghai 200025, China; tjyt83@163.com (Y.T.); xsz40689@rjh.com.cn (S.X.); 2 Department of Clinical Laboratory, Shanghai Sixth People’s Hospital Affiliated to Shanghai Jiao Tong University School of Medicine, Shanghai 200233, China; joanwu29@hotmail.com (Q.W.); winniewin@163.com (Y.W.); wangjq550318@sjtu.edu.cn (J.W.); 3 Department of Laboratory Medicine, Taicang Loujiang New City Hospital (Taicang Branch of Ruijin Hospital Affiliated with Shanghai Jiao Tong University School of Medicine), Taicang 215400, China

**Keywords:** bloodstream infection, *Escherichia coli*, genome epidemiology, ARGs, plasmid

## Abstract

**Background**: Bloodstream infection (BSI) caused by *Escherichia coli* has become a growing clinical threat due to its high incidence and mortality rates. Despite its significant global health burden, the genomic diversity of *E. coli* in Shanghai has not been systematically investigated, with ramifications for treatment and prevention. This study aimed to assess the prevalence and genomic epidemiology of *E. coli* BSI isolates in Shanghai, delineate predominant lineages and their related phenotypic and genomic characteristics, and elucidate the population structure. **Methods**: A total of 136 *E. coli* strains were collected from a tertiary hospital in Shanghai between 2023 and 2024. We conducted comprehensive analyses, including multilocus sequence typing (MLST), core genome MLST (cgMLST), clonal complex assignment, serotype prediction, identification of antimicrobial resistance genes (ARGs) and plasmid-borne virulence genes, and plasmid mobility prediction. **Results**: The predominant clonal complex was CC131 (22.8%), followed by CC14 (14%). The highest resistance rate was observed for ampicillin (85.3%), whereas amikacin (3.7%), ertapenem (2.9%), imipenem (2.9%), minocycline (2.2%), and meropenem (2.2%) retained excellent in vitro activity. High resistance to ciprofloxacin (63.2%) and levofloxacin (62.5%) was observed, primarily mediated by mutations in *gyrA* (S83L, D87N) and *parC* (S80I), particularly in ST1193 strains. Plasmid-borne siderophore virulence genes co-occurred as gene modules, the majority of which were located on transferable IncFIB plasmids. However, this trend was not uniform across lineages, with the CC131 clones predominantly carrying mobilizable plasmids, while CC14 clones predominantly harbored non-mobilizable plasmids. **Conclusions**: Our findings reveal the dynamic genetic diversity of *E. coli* BSI isolates at a tertiary hospital in Shanghai, highlighting the importance of genomic surveillance and providing valuable insights for infection control.

## 1. Introduction

Bloodstream infection (BSI) is the intermittent or persistent presence of pathogenic microorganisms in the blood, causing bodily harm, systemic inflammation, and potentially leading to shock, multiple organ failure, and death [1]. The public health burden of BSI has intensified in recent years, driven by an ageing population, increased use of invasive medical procedures, and the continued spread of drug-resistant bacteria [2]. The global annual incidence of BSI is approximately 150 cases per 100,000 population, with a 30-day mortality rate of approximately 17% [2]. These rates vary considerably by geographic region and level of national development. In China, the average annual incidence of BSI was 369 cases per 100,000 population, with a 28-day mortality rate of around 25% [3], substantially higher than the global average. Additionally, a study focusing on ICU patients in Shanghai reported an incidence rate of approximately 10.11% and a corresponding mortality rate of about 35.21% [4], highlighting the severe burden of BSI in critical care settings. However, despite its clinical relevance, specific population-based epidemiological data on *E. coli* BSI in Shanghai remain scarce, underscoring the need for this genomic investigation.

*Escherichia coli* is the most common Gram-negative pathogen causing BSI and is associated with the highest 30-day mortality among such infections [2]. It exhibits high rates of drug resistance and a broad resistance spectrum, thereby limiting treatment options and adversely affecting clinical outcomes for patients. Genomic epidemiological surveillance enables the characterization of antimicrobial resistance, virulence, environmental adaptability, and transmission dynamics of *E. coli*, providing critical insights that support infection control efforts and improve patient prognoses.

*E. coli* ST131 is a globally prevalent clonal group that was identified in clinical samples in the early 2000s, though it was not formally recognised as a global pandemic clone with high transmissibility until 2008. ST131 is the principal lineage responsible for global fluoroquinolone resistance (FQR), primarily causing bloodstream and urinary tract infections [5]. This lineage is commonly associated with the production of extended-spectrum β-lactamases (ESBLs), particularly *blaCTX-M-15* [6]. ST131 exhibits considerable genetic diversity and is a major cause of both healthcare-associated and community-acquired *E. coli* infections [7]. In contrast, *E. coli* ST1193 has emerged as a globally recognised high-risk clonal group. Previous studies have shown that ST1193 is characterized by high conservation, multidrug resistance, and substantial virulence [8]. Notably, high-level quinolone resistance is a defining feature of ST1193, which is predominantly associated with community-acquired *E. coli* infections [9,10]. Despite extensive global research on these two clonal types, studies of their genomic prevalence, genomic diversity, resistance profiles, and virulence landscapes in Shanghai remain limited, and further investigation is needed. To elucidate the prevalence of BSI *E. coli* and identify the dominant clonal types in Shanghai, we conducted genomic epidemiological analyses of *E. coli* isolates collected from a tertiary hospital in Shanghai. Our study investigated whether ST131 and ST1199 represent the predominant clones and the factors driving their prevalence.

Virulence is a critical determinant of *E. coli* survival and pathogenicity, significantly influencing bacterial dissemination in the bloodstream. Among numerous virulence factors, iron-acquisition systems are regarded as vital determinants of virulence because iron limitation is a significant selective pressure in the host environment [11]. Plasmids are the primary vectors for horizontal gene transfer, facilitating the rapid dissemination of antimicrobial resistance and virulence genes across diverse strains and species, thereby enhancing bacterial pathogenicity and resistance [12]. To elucidate the impact of plasmid-mediated synergistic mechanisms linking virulence and drug resistance on the pathogenic potential of *E. coli*, we investigated the distribution and coexistence of virulence genes on plasmids, as well as the associated risks of plasmid-mediated horizontal transfer.

Given the threat posed by BSI in healthcare settings, this study aimed to systematically elucidate the genomic features of BSI *E. coli* in Shanghai using whole-genome sequencing and multidimensional bioinformatics analyses. A comprehensive analysis was conducted to assess the dominance of predominant clones, their genomic diversity, resistance profiles, and features of plasmid-borne virulence genes. Our studies indicated that ST131 is the predominant clone, exhibiting significant genetic variation, whereas ST1193 is the second most prevalent clone, characterized by genetic conservation. Notably, the majority of the virulence plasmids carried by ST131 were predicted to be mobilizable, while most of the virulence plasmids carried by ST1193 were predicted to be non-mobilizable. Plasmid-borne virulence genes exhibited a lineage-specific pattern. In contrast, plasmid-borne resistance genes disseminated widely and were not lineage-dependent.

## 2. Results

### 2.1. Clinical Characteristics of Patients

A total of 136 unique isolates were included, and the related clinical characteristics are shown in Table 1. We analysed the patients’ gender, age, comorbidities at admission, invasive procedures, and medication history within 90 days preceding the BSI. Patient ages ranged from 18 to 101 years, with a median of 69. Hospital-acquired BSI occurred in 86 patients (63.2%). Among the 82 (60.3%) cases with known infection sources, urinary tract infections were most common (36/82, 43.9%), followed by skin and soft tissue infections (20/82, 24.4%). The most prevalent underlying condition at admission was hypertension (44.1%). Among invasive procedures performed within 90 days before BSI onset, arterial catheterization was most common (44.9%), and 118 patients (86.8%) had received antibiotics.

### 2.2. Antimicrobial Susceptibility Testing for 136 Strains

The antimicrobial susceptibility profiles of the 136 *E. coli* isolates against 22 antibiotics are shown in Figure 1. The highest resistance rate was observed for AMP (85.3%), while lower resistance rates were observed for AK (3.7%), ETP, IPM (2.9%), and MH, MEM (2.2%). Among all, 114 strains (83.8%) were classified as multidrug-resistant (MDR).

### 2.3. Concordance Between Phenotypic and Genotypic Resistance Profiles

To assess the correlation between resistance genes and phenotypic resistance, we evaluated concordance between genotypes and phenotypes for 22 antibiotics. We classified the 22 antibiotics for susceptibility testing into 5 antibiotic categories, while the identified resistance genes correspond to 12 antibiotic classes (Table 2).

The resistance phenotypes and genetic characteristics of 136 *E. coli* strains are shown in Table 2, and the concordance between resistance phenotypes and genotypes is shown in Table 3. Concordance was almost perfect for nine antibiotics (KZ, CRO, IPM, ETP, GEN, SXT, CIP, LEV, MEM), with kappa values ranging from 0.81 to 1.00. In contrast, five antibiotics (CXM, SAM, FOX, AMP, AMC) showed substantial agreement, with kappa values between 0.61 and 0.80. Three antibiotics (FEP, ATM, TOB) showed moderate agreement, with kappa values from 0.41 to 0.60. Four antibiotics (CAZ, TZP, AK, TET) showed fair agreement, with kappa values ranging from 0.21 to 0.40. However, MH had a kappa value of only 0.124, and the detection rate of its predicted resistance genotype was higher than that of its resistance phenotype.

### 2.4. MLST, CC, and cgMLST Reveal the Genetic Diversity of E. coli

MLST analysis of 136 *E. coli* isolates revealed 45 known STs, among which ST131 was predominant (22.8%), followed by ST1193 (14%) (Figure 2a). Among the 45 STs detected, 26 were classified into 18 clonal complexes (CCs), while the remaining 19 were not classified into any CC. Six ST types, including ST48, ST18520, ST744, ST215, ST207,and ST10, all belong to CC10. CC131 and CC14 were the dominant groups (Figure 2c).

The cgMLST analysis identified eleven genetically distinct HC10 clusters, encompassing 23 isolates (Figure 2b), which shared highly similar core-genome profiles, indicating close genetic relatedness within this subset. Of these 23 clustered isolates, 12 belonged to ST131 and were distributed across six HC10 clusters, highlighting ST131’s predominance among genetically related isolates in this cohort. Notably, ST131’s presence across multiple distinct HC10 clusters also reflects substantial genomic diversity within this lineage. The remaining 113 isolates did not fall into any HC10 cluster, suggesting limited evidence of highly related genotypes within the study cohort under the defined threshold.

### 2.5. Genetic Lineages of E. coli Revealed by the Phylogenetic Tree

The maximum likelihood phylogenetic tree showed that the 136 genomes were divided into four main clades. Group B2 was the most prevalent phylogroup in our study (47.1%), followed by group D (16.9%), while group E had the lowest detection rate (2.2%). The most dominant STs, ST131 and ST1193, both belong to group B2. 35 distinct O-antigen serotypes were identified. O25 was the predominant serotype (22.8%), followed by O75 (8.8%), with nine strains non-typable. Only four isolates were found to possess carbapenemase genes, specifically *blaKPC_2* (0.7%) and *blaNDM_5* (2.2%). Notably, none of the isolates in this study contained two distinct carbapenemase genes simultaneously (Figure 2c). Phenotypic testing confirmed that all four gene-positive isolates produced functional carbapenemases (Figure S1).

### 2.6. Sublineage Characterization of ST131 Isolates

To further characterize the dominant ST131 clone, we performed a refined subtyping analysis of our ST131 isolates. As shown in Figure 3, the majority of ST131 isolates belonged to the *fimH30* sublineage (Clade C/H30). Within this clade, the H30Rx subclone (Clade C2) was predominant, and Virotype C was the most frequently identified virotype among these isolates.

### 2.7. Antibiotic Resistance Genes Profile

A total of 68 ARGs were detected in these 136 clinical strains (Figure 4). Among the 20 Ambler classes of β-lactamase genes, 15 belong to class A, 1 to class B, 2 to class C, and 2 to class D. ST131 is a globally prevalent clone strongly associated with *blaCTX-M-15*. In our study, *blaCTX-M-15* was found in 19 isolates, 15 (79%) of which belonged to ST131. *BlaCTX-M-27* was found in 17 isolates, 12 (71%) of which were classified as ST131. *BlaOXA-1* was found in 15 isolates, 13 (87%) of which belonged to ST131. Interestingly, only two ST131 isolates possessed all three resistance genes simultaneously.

Fifteen unique aminoglycoside resistance genes were detected. 15 strains carried *aac(6′)-Ib-cr*, 13 of which (87%) belonged to ST131. Four genes encoding resistance to tetracyclines were identified. The most prevalent of these was *tet(A)* (38%), which was present across different STs. The fosfomycin resistance gene *fosA3* and the lincosamide resistance gene *lnu(F)* were detected across different STs at frequencies of 10% and 7%. The polymyxin resistance gene *mcr-1.1* was found in four strains belonging to different STs.

Six quinolone resistance genes were detected, with *oqxA* and *oqxB* co-occurring in pairs (*n* = 3). All quinolone resistance genes identified in this study (*oqxA*, *oqxB*, *qnrB1*, *qnrB4*, *qnrS1*, *qnrS2*) were plasmid-mediated and conferred low-level quinolone resistance. Furthermore, mutations in the quinolone resistance-determining regions (QRDRs) were analysed (Figure S2). A total of 76 isolates harboured double *gyrA* mutations (S83L and D87N) in combination with the *parC*_S80I mutation. This combination mediates high-level quinolone resistance. Notably, all 19 ST1193 isolates exhibited double *gyrA* mutations (S83L and D87N) along with *parC*_S80I and *parE*_L416F mutations. Moreover, all of the ST410 (*n* = 4), ST405 (*n* = 3) isolates harboured *gyrA*_S83L, *gyrA*_D87N, *parC*_S80I, and *parE*_S458A mutations. The two ST457 isolates displayed *gyrA*_S83L, *gyrA*_D87Y, *parC*_S80I, and *parE*_S458A mutations. Similarly, the two ST744 isolates contained *gyrA*_S83L, *gyrA*_D87N, *parC*_S80I, and *parC*_A56T mutations.

### 2.8. Plasmids and Plasmid-Borne Virulence Genes

Further analysis revealed differences in the distribution of predicted plasmids and plasmid-associated virulence genes among different clonal lineages. A total of 606 putative plasmids were reconstructed in 98% of isolates. However, only 190 plasmids, found in 118 isolates, were predicted to carry 54 virulence genes (Figure 5). Plasmids predicted to carry virulence genes were classified into 19 replicon types, with IncFIB the dominant type. IncFIB plasmids were found in 86/136 strains (63%) and were distributed across different STs. Notably, the carriage rate of IncFII replicons was significantly higher in ST1193. Among the 22 strains predicted to carry an IncFII replicon, 13 (59%) belonged to the ST1193 sequence type.

Virulence genes and replicon types co-occurred within the same predicted plasmid contigs. All strains harbouring *aatA* (*n* = 3/136) also carried IncFIB and IncHI1B replicons. In contrast, strains carrying *ce1a* (*n* = 8/136) harboured the IncFIB and rep_cluster_2350 replicons. Similarly, all strains with *cma* (*n* = 15/136) carried IncFIB replicons. Additionally, strains harbouring *EcSMS35_B0007* (*n* = 3/136) carried the IncFIB replicon. Strains with *mcba* (*n* = 2/136) carried IncFIA and IncFIC replicons. Furthermore, strains carrying *pECS88_0104* (*n* = 4/46) consistently harboured the IncFIB replicon. *Ce1a*, *aatA*, *cma*, *EcSMS35_B0007*, *mcba* and *pECS88_0104* were found in various STs.

Three genetic modules were predicted in plasmids. They differed in genetic composition and replicons. Module 1 consisted of *iroB*, *iroC*, *iroD*, *iroE*, and *iroN*, and was found in 31 plasmids. 68% (*n* = 21) of Module 1 harboured the IncFIB replicon and were carried by putatively transferable plasmids (including both conjugative and mobilizable types). Module 2 was composed of *iucA*, *iucB*, *iucC*, *iucD*, and *iutA*, and was detected in 49 plasmids. Among the plasmids carrying Module 2, 47% (*n* = 23) were predicted to be transferable, while the remaining 53% (*n* = 26) were putatively non-transferable. Module 3 consisted of *sitA*, *sitB*, *sitC*, and *sitD*, and was found in 24 plasmids. 54% (*n* = 13) of plasmids harbouring Module 3 carried the IncFIB replicon and were predicted to be transferable. The three genetic modules were distributed across diverse STs without host preference and were involved in iron acquisition.

### 2.9. Plasmids Are Enriched in the Predominant CC131 and CC14 Clones

The mobility prediction classified 62% (118/190) of plasmids as transferable, including 51% (97/190) conjugative plasmids and 11% (21/190) mobilizable plasmids that rely on conjugative plasmids for transfer. Non-transferable plasmids accounted for 38% (72/190). *TraJ* and *traT* have been described as transfer-associated genes frequently found in conjugative plasmids [13,14]. Similarly, our study predicted that 74 (76%) conjugative plasmids carried at least one of these two genes (Figure 6a). The distribution of predicted mobile versus non-mobilizable plasmids differed significantly across clonal complexes (Figure 6b). The analysis of plasmid distribution across *E. coli* CC revealed that CC131 strains had the highest average plasmid burden. Most plasmids from CC131 strains (75%, *n* = 30/40) were predicted to be transferable. In contrast, most plasmids from CC14 strains (75%, *n* = 18/24) were predicted to be non-transferable. This difference was statistically significant (Fisher’s exact test, *p* < 0.001), indicating that CC131 harbors a substantially higher proportion of mobilizable plasmids than CC14 (Figure 6c).

## 3. Discussion

Our study aimed to acquire genomic and phenotypic epidemiological data of *E. coli* bloodstream isolates in Shanghai over two years, revealing novel insights into genetic diversity.

In this study, ST131 was the most prevalent lineage during 2023–2024, consistent with the findings reported by the University of Michigan Medical School [2]. Previous research has found that ST131 isolates associated with urosepsis increased mortality [15]. In alignment with this, our study found that ST131 accounted for 57.1% of fatal cases, with five of these fatalities arising from bloodstream isolates linked to urinary tract infections, underscoring the pathogenic potential of ST131 strains. However, some studies have not found a significant association between ST131 and BSI mortality [16,17]. The high pathogenicity of ST131 is mainly linked to its possession of numerous virulence genes, strong biofilm-forming capacity, and high transmissibility [18]. The higher plasmid transferability observed in CC131 is concerning, as it implies a greater potential for the horizontal spread of antimicrobial resistance genes to other pathogenic or commensal *E. coli* strains. This may partially explain the predominance of CC131 in clinical settings and its association with multidrug-resistant infections. These findings underscore the clinical significance of ST131 and warrant vigilance given its poor prognosis.

The observed phenotypic resistance did not fully align with genotypic profiles, highlighting the diversity and complexity of antimicrobial resistance mechanisms. Notably, poor concordance between genotype and phenotype was observed for ceftazidime, piperacillin–tazobactam, amikacin, and minocycline, with the prevalence of resistance genes exceeding phenotypic resistance. Previous reports suggest that CTX-M β-lactamases frequently demonstrate inadequate hydrolytic activity against ceftazidime, which is not enough to cause clinical resistance [19]. Furthermore, CTX-M enzymes were more readily inhibited by β-lactamase inhibitors, such as tazobactam [19,20]. This could potentially explain the observed susceptibility to CAZ and TZP in a subset of *E. coli* isolates despite the presence of resistance genes. Moreover, *tet(B)* not only mediates the active efflux of minocycline but also degrades it, representing the primary mechanism of minocycline resistance in *E. coli* [21]. Ribosomal protection mediated by *tet(M)* constitutes a significant mechanism of minocycline resistance [22]. However, most *tet(B)*-harbouring isolates were susceptible to minocycline but resistant to tetracycline in this study. This finding indicated that the selective pressures exerted on the *tet(B)*-encoded efflux pump differ between these two antibiotics. One possible explanation for this phenomenon is that minocycline’s enhanced lipophilicity facilitates greater membrane penetration and intracellular accumulation [23]. In contrast, the *tet(B)* efflux pump shows lower affinity for minocycline, resulting in less effective extrusion than with tetracycline. In our study, most *E. coli* isolates harboring *aac(6′)-Ib-cr* remained susceptible to amikacin, which could potentially be attributable to insufficient expression of the AAC(6′)-Ib-cr enzyme to confer phenotypic resistance [24]. Conversely, phenotypic resistance to tetracycline was observed in a subset of isolates despite the absence of known tetracycline resistance genes; this may be attributed to multidrug efflux pump overexpression and porin-mediated permeability changes [25], though these mechanisms were not experimentally assessed here. The discordance between resistance genotype and phenotype underscores the need for antimicrobial selection to extend beyond genotypic detection. It is essential that phenotypic susceptibility testing be used to inform appropriate antibiotic choices and ensure precise therapy for patients.

Our findings suggest that the capacity for iron acquisition may be acquired through horizontal gene transfer between strains, potentially contributing to bacterial virulence and systemic infection. However, this interpretation remains hypothetical in the absence of functional experimental validation. Several studies have demonstrated that deleting genes linked to iron acquisition systems in extraintestinal pathogenic *E. coli* (EXPEC), specifically *iroBCDEN* and *sitABCD*, significantly diminishes virulence in infection models [26,27]. These results suggest a strong correlation between iron-acquisition virulence genes and high-virulence phenotypes, highlighting the essential role of iron uptake capacity in bacterial survival and pathogenicity.

Another significant finding of our study is that the difference in plasmid transferability between CC131 and CC14 suggests fundamentally distinct evolutionary trajectories in horizontal gene transfer (HGT) capacity. CC131 appears to have maintained or acquired a larger repertoire of mobilizable plasmids, which may facilitate rapid dissemination of resistance and virulence determinants within this lineage. In contrast, most plasmids in CC14 were predicted to be non-transferable. This result is consistent with the highly conserved genetic characteristics of CC14 and indicates that its virulence genes were acquired primarily through clonal expansion and vertical transmission, rather than through horizontal transfer between lineages. A signature feature of CC14 is its high-level quinolone resistance, driven predominantly by multiple point mutations in the chromosomal QRDRs. Notably, the double mutation in *gyrA* (S83L, D87N) and the *parC*_S80I substitution are central to this resistance [28]. The substitutions S83L and D87N are located within the QRDR of the GyrA subunit of DNA gyrase, which spans amino acid residues 67 to 106 in *E. coli*, and this region is near the catalytic tyrosine residue (Tyr122) that covalently binds DNA during strand breakage and rejoining [29]. The QRDR forms part of the quinolone-binding pocket, where fluoroquinolones interact with the gyrase-DNA complex to block DNA resealing. GyrA proteins carrying these substitutions display lower binding affinities for quinolones than the wild type, and these mutations are known to synergize with *parC* alterations to produce high-level fluoroquinolone resistance [30]. ST1193 isolates in Shanghai during 2019–2020 carried mutations in *gyrA* (S83L, D87N) and *parC* (S80I, E84V) [31], whereas all ST1193 isolates in this study harboured the *gyrA* (S83L, D87N), *parC* (S80I), and *parE*_L416F mutations, which confer quinolone resistance. The QRDR mutation profiles therefore differed between the current isolates and previously reported Shanghai isolates. These findings may reflect temporal changes in circulating ST1193 genotypes. However, this observation alone is insufficient to infer lineage evolution. Further longitudinal and phylogenetic analyses are needed to determine whether these changes represent true lineage evolution.

Infections caused by *E. coli* pose a significant clinical challenge in healthcare settings. A considerable proportion of isolates exhibit multidrug resistance, limiting therapeutic options. This resistance stems from a highly adaptable genetic architecture that contains numerous mobile genetic elements, enhancing bacterial adaptation to antibiotics and environmental pressures and conferring novel resistance and virulence traits. We investigated the molecular mechanisms underlying antibiotic resistance and the plasmid-borne virulence gene profiles of *E. coli* isolates from BSI. Our analysis revealed a predominance of ST131 and ST1193 and highlighted the contrasting genomic features, particularly in plasmid transferability, between these two dominant clonal complexes. These findings underscore the differential dissemination potential of resistance and virulence determinants among *E. coli* lineages, which may have implications for infection control and antimicrobial stewardship in managing *E. coli* BSI.

This study has several limitations. First, the small sample size limited our ability to analyse the risk factors for the outcome. The association between ST131 and mortality was based on a univariate analysis and could be confounded by age, comorbidities, infection source, hospital acquisition, and treatment. Due to the limited number of fatal events (*n* = 14), a multivariable logistic regression adjusting for these covariates was not statistically feasible. Therefore, the observed association should be interpreted with caution, and residual confounding cannot be excluded. Second, plasmid reconstruction was performed using short-read whole-genome sequencing data. Therefore, the predicted plasmid mobility in these isolates should be considered hypothesis-generating and requires experimental validation to confirm the actual mobility potential. Third, this study was limited to a 2-year collection period and therefore could not assess long-term evolutionary trends or clonal turnover beyond this timeframe. Continuous, long-term genomic surveillance is warranted for early detection of high-risk clone dissemination and emerging resistance mechanisms. Fourth, our current study primarily focused on acquired resistance genes and lacked transcriptomic or proteomic data to quantify efflux pump or porin expression levels. Fifth, our analysis did not include mobile genetic elements other than plasmids. Future research will use long-read whole-genome sequencing to validate the complete plasmid structure, characterize the resistance and virulence genes they harbour, and examine insertion sequences associated with plasmids. This will enable us to investigate the impact of insertion sequence-mediated gene disruption on bacterial antimicrobial resistance. Sixth, because we lack detailed spatio-temporal patient data, we cannot distinguish true clonal dissemination from the concurrent circulation of genetically related strains. Therefore, HC10 genetic clusters indicate close genomic relatedness but do not constitute evidence of recent in-hospital transmission. Conversely, the absence of HC10 clusters does not exclude recent clonal expansion, particularly given the fixed nature of the HC10 threshold and the single-center sampling design. Our cgMLST analysis should be interpreted as a descriptive survey of genetic diversity rather than a transmission dynamics investigation.

Despite these limitations, our genomic characterization of ST131 and ST1193 provides valuable insights into the molecular epidemiology of these high-risk lineages.

## 4. Materials and Methods

### 4.1. Strain Isolation

A total of 136 *E. coli* isolates were collected from blood culture–positive patients at the Clinical Laboratory of Shanghai Sixth People’s Hospital between January 2023 and December 2024. After isolating and purifying single colonies, species identification was performed using matrix-assisted laser desorption/ionization time-of-flight mass spectrometry (MALDI-TOF MS; Bruker Daltonics GmbH, Bremen, Germany) [32].

### 4.2. Antimicrobial Susceptibility Testing

A total of 136 non-duplicate *E. coli* isolates were tested for susceptibility to 22 antimicrobial agents across 5 classes. Ampicillin, cefazolin, cefuroxime, ceftriaxone, ciprofloxacin, levofloxacin, tetracycline, compound sulfamethoxazole (SMZ-TMP), gentamicin, cefepime, aztreonam, tobramycin, ceftazidime, ampicillin/sulbactam, amoxicillin/clavulanate, cefoxitin, amikacin, piperacillin/tazobactam, ertapenem, imipenem, minocycline, and meropenem were tested by the disk diffusion method on Mueller–Hinton agar plates (Oxoid, Basingstoke, UK). The susceptibility results were interpreted according to the guidelines of the Clinical and Laboratory Standards Institute (CLSI, M100). *E. coli* ATCC 25922 (American Type Culture Collection, Manassas, VA, USA) was used as a quality control organism. Inoculated Mueller-Hinton agar plates were incubated at 35 ± 2 °C in ambient air for 16–18 h, The resulting zone diameters were interpreted according to the CLSI M100-Ed35 (2025) breakpoints. The isolate was categorized as MDR if it was non-susceptible to at least one agent in three or more antimicrobial categories [33]. The following 13 antimicrobial categories were included in the classification based on the AST panel tested: penicillins; penicillins + β-lactamase inhibitors; cephalosporins (1st, 2nd, 3rd, and 4th generations); cephamycins; carbapenems; monobactams; aminoglycosides; fluoroquinolones; sulfonamides; and tetracyclines.

### 4.3. Whole-Genome Sequencing Analysis and Bioinformatics Analyses

A total of 136 *E. coli* strains were subjected to whole-genome sequencing. Genomic DNA was sequenced on the Illumina NovaSeq platform in paired-end mode (2 × 150 bp).

MLST and CC were confirmed using the MLST database (https://pubmlst.org/organisms/escherichia) (accessed on 13 July 2026). Minimal spanning tree and cgMLST bubble plot were generated using the Enterobase platform v1.2.0 (https://enterobase.warwick.ac.uk) (accessed on 15 July 2026) [34]. Phylotypes and serotypes were detected via (http://cloud2.mimazi.net:9001/member/index.html) [35,36] (accessed on 15 July 2026). The whole genome was annotated with Prokka v1.14.6 [37]; the core genomes were extracted and compared with Roary v3.13.0 [38]; and a phylogenetic tree based on core genes was constructed with FastTree v2.1.10 [39]. The phylogenetic tree was uploaded to the ITOL website v7.6 (https://itol.embl.de/) (accessed on 17 July 2026) for visualization, and the midpoint rooting method was used to root it. MOB-recon v3.1.9 was used to cluster and reconstruct plasmid sequences, and MOB-typer v3.1.9 was applied to assign plasmid types, identify replicon types, and predict the conjugative potential of the plasmids [40]. Antimicrobial resistance genes (ARGs) and virulence genes were predicted using ABRicate v1.0.1 with the ResFinder and Ecoli_VF databases. *FimH* alleles and ST131 subclonal assignment were performed using ST131Typer v1.0.0 (https://github.com/JohnsonSingerLab/ST131Typer) (accessed on 15 July 2026) [41]. Isolates were assigned to sublineages according to the ST131Typer output. The virotype of each ST131 isolate was detected using VirulenceFinder v3.2.1 (https://genepi.food.dtu.dk/virulencefinder) (accessed on 15 July 2026). MOB-recon, MOB-typer, ResFinder, Ecoli_VF, and VirulenceFinder applied minimum thresholds of 95% nucleotide identity and 90% coverage.

We analysed the cgMLST profiles of the *E. coli* isolates based on 2513 core genome loci, and then performed hierarchical clustering at level 10 (HC10) to define genetically closely related strains. Strains differing by ≤10 cgMLST alleles were considered genetically similar [42]. A gene module is defined as a set of co-occurring gene clusters [43]. These genes are consistently co-located on the same plasmid and are always present or absent together across strains. Our co-occurrence analysis is based on presence/absence data of virulence genes, rather than on transcriptional co-expression.

### 4.4. Concordance Between Phenotypic and Genotypic Resistance Profiles

Cohen’s Kappa coefficient was used to evaluate the concordance between the observed antimicrobial susceptibility phenotype and predicted genotype. The Kappa values were interpreted according to the Landis & Koch criteria: 0.81–1.00 was almost perfect agreement, 0.61–0.80 was substantial, 0.41–0.60 was moderate, 0.21–0.40 was fair, <0.20 was slight, and ≤0 was no agreement [44]. The 95% confidence interval (CI) for kappa was calculated as κ ± 1.96 × SE(κ), where SE(κ) represents the standard error of the kappa coefficient. Both sensitive and intermediate phenotypes are considered sensitive for analytical purposes.

### 4.5. Phenotypic Detection of Carbapenemase Production

Carbapenemase production was detected using the NG-Test CARBA 5 immunochromatographic assay according to the manufacturer’s instructions.

### 4.6. Statistical Analysis

Continuous variables are presented as median with interquartile range (IQR); categorical variables are presented as counts and percentages (*n*, %). For categorical comparisons, Fisher’s exact test was used when expected cell counts were less than 5. When the contingency table was larger than 2 × 2 and the number of observations was limited, Fisher’s exact test with Monte Carlo simulation was applied to obtain accurate *p* values. A two-sided *p* value < 0.05 was considered statistically significant. SPSS version 29.0 (IBM, Armonk, NY, USA) was used for statistical analysis. The odds ratio (OR) with 95% confidence interval (CI) was calculated to estimate the strength of association between predominant sequence type and 30-day mortality.

## 5. Conclusions

This study reveals the genomic characteristics of *E. coli* BSI within a single tertiary care center in Shanghai, highlighting the local predominance of CC131 and CC14. Our study emphasises the antimicrobial resistance profiles of *E. coli* strains and the characteristics of plasmid-mediated virulence genes, as well as the reasons for the inconsistency between resistance phenotypes and genotypes, thereby providing effective strategies for the prevention and control of *E. coli* infections. However, further multi-center surveillance is warranted to confirm the broader epidemiological landscape across Shanghai.

## Figures and Tables

**Figure 1:**
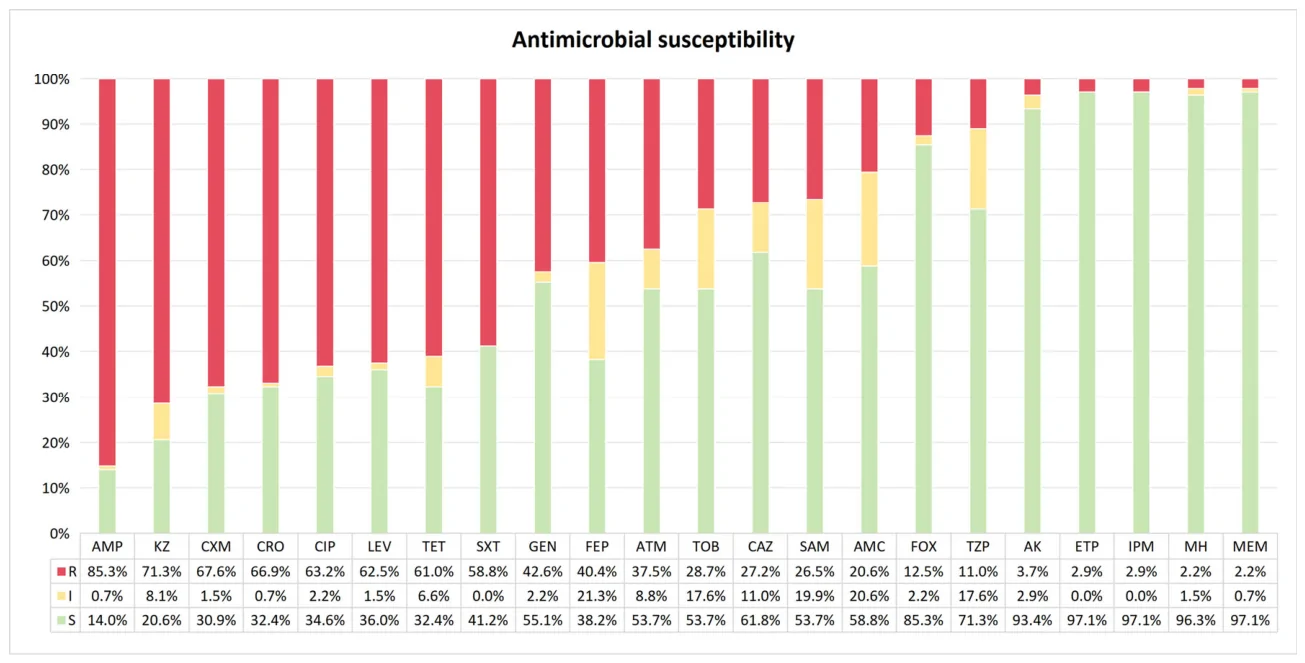
Antimicrobial susceptibility of 136 *E. coli* strains to 22 clinically relevant antibiotics. R, resistant; I, intermediate; S, susceptible; AMP, Ampicillin; KZ, Cefazolin; CXM, Cefuroxime; CRO, Ceftriaxone; CIP, Ciprofloxacin; LEV, Levofloxacin; TET, Tetracycline; SXT, Compound Sulfamethoxazole (SMZ-TMP); GEN, Gentamicin; FEP, Cefepime; ATM, Aztreonam; TOB, Tobramycin; CAZ, Ceftazidime; SAM, Ampicillin/Sulbactam; AMC, Amoxicillin/Clavulanate; FOX, Cefoxitin; TZP, Piperacillin/Tazobactam; AK, Amikacin; ETP, Ertapenem; IPM, Imipenem; MH, Minocycline; MEM, Meropenem.

**Figure 2:**
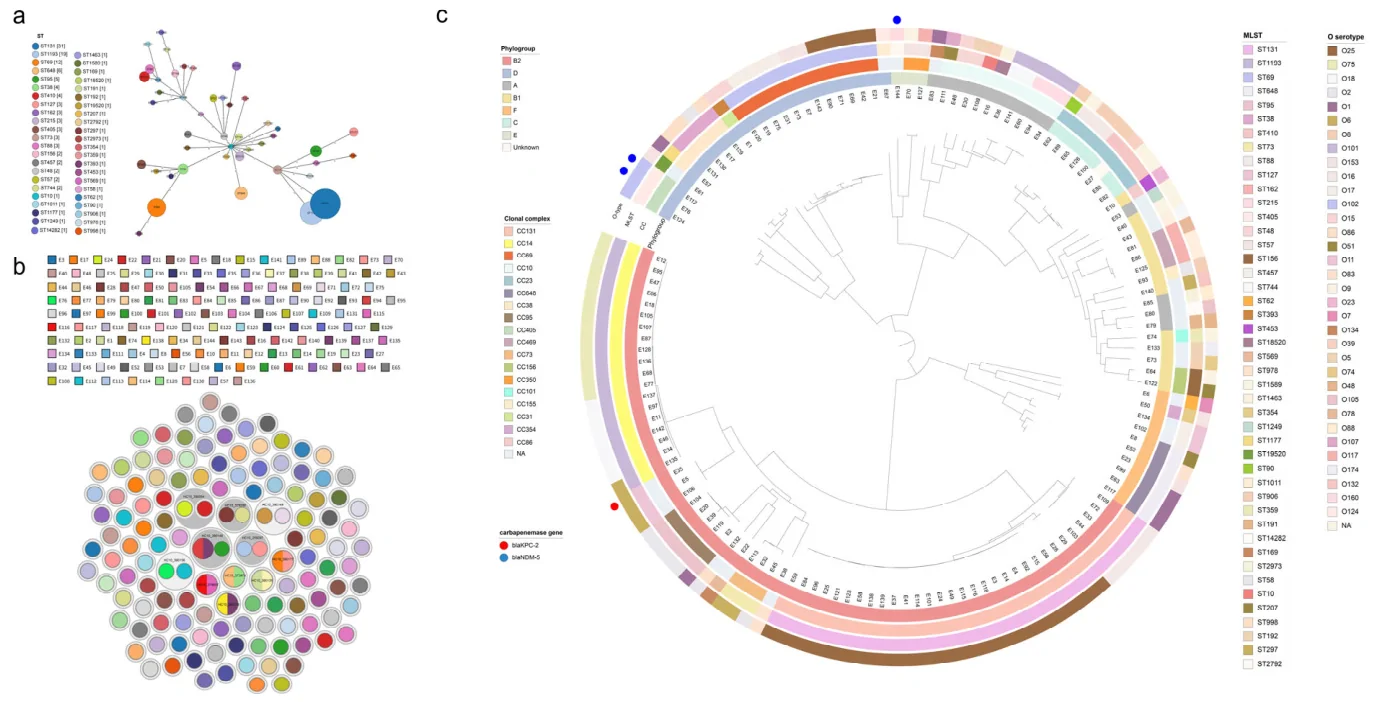
Genotypic relationship revealed by MLST, cgMLST, and phylogenetic analysis. (**a**) Smallest spanning tree based on MLST for 136 *E. coli* strains. The circular node represents different STs, and the node diameter is proportional to the number of clinical isolates. (**b**) Bubble plot of genetic structure based on 2513 core genome loci. Strains with HC ≤ 10 were clustered into a bubble, revealing genetically similar strains that differ by ≤10 cgMLST alleles. (**c**) Midpoint-rooted phylogenetic tree of 136 *E. coli* strains annotated with phylogenetic lineage, clonal complex, sequence type, O-antigen serotype, and carbapenemase carriage.

**Figure 3:**
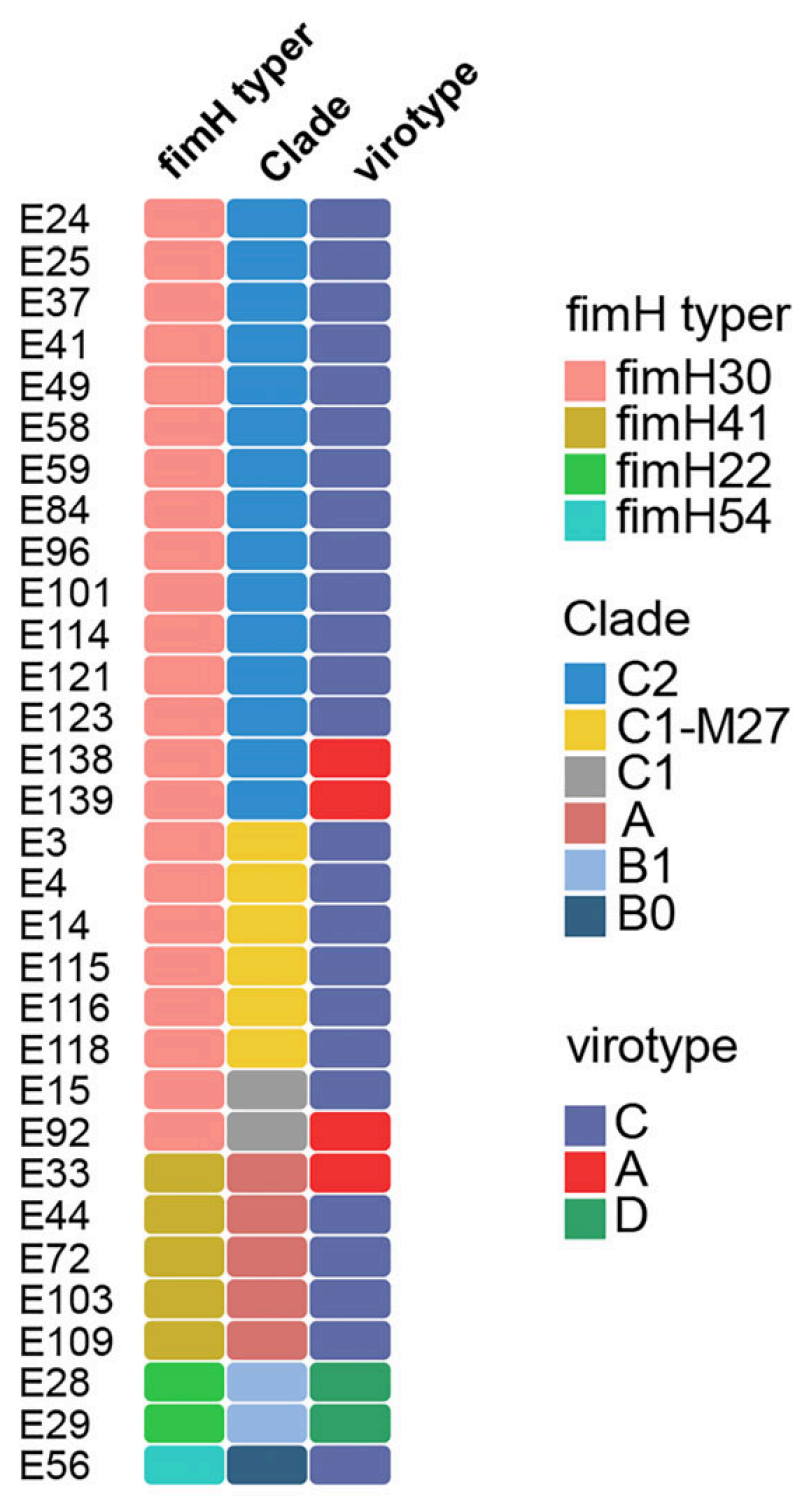
FimH alleles, H30 subclade assignment, and virotype determination of ST131 isolates. Four *fimH* types were detected, including *fimH30* (23/31, 74.2%), *fimH41* (5/31, 16.1%), fimH22 (2/31, 6.5%), and *fimH54* (1/31, 3.2%). Among *fimH30*-positive strains, Clade C2 accounted for 65.2% (*n* = 15/23), compared with 34.8% (*n* = 8/23) for Clade C1 (including 6 C1-M27). Virotype C was the most prevalent among our ST131 strains (80.6%, 25/31), followed by virotype A (12.9%, 4/31), with virotype D the least prevalent (6.5%, 2/31).

**Figure 4:**
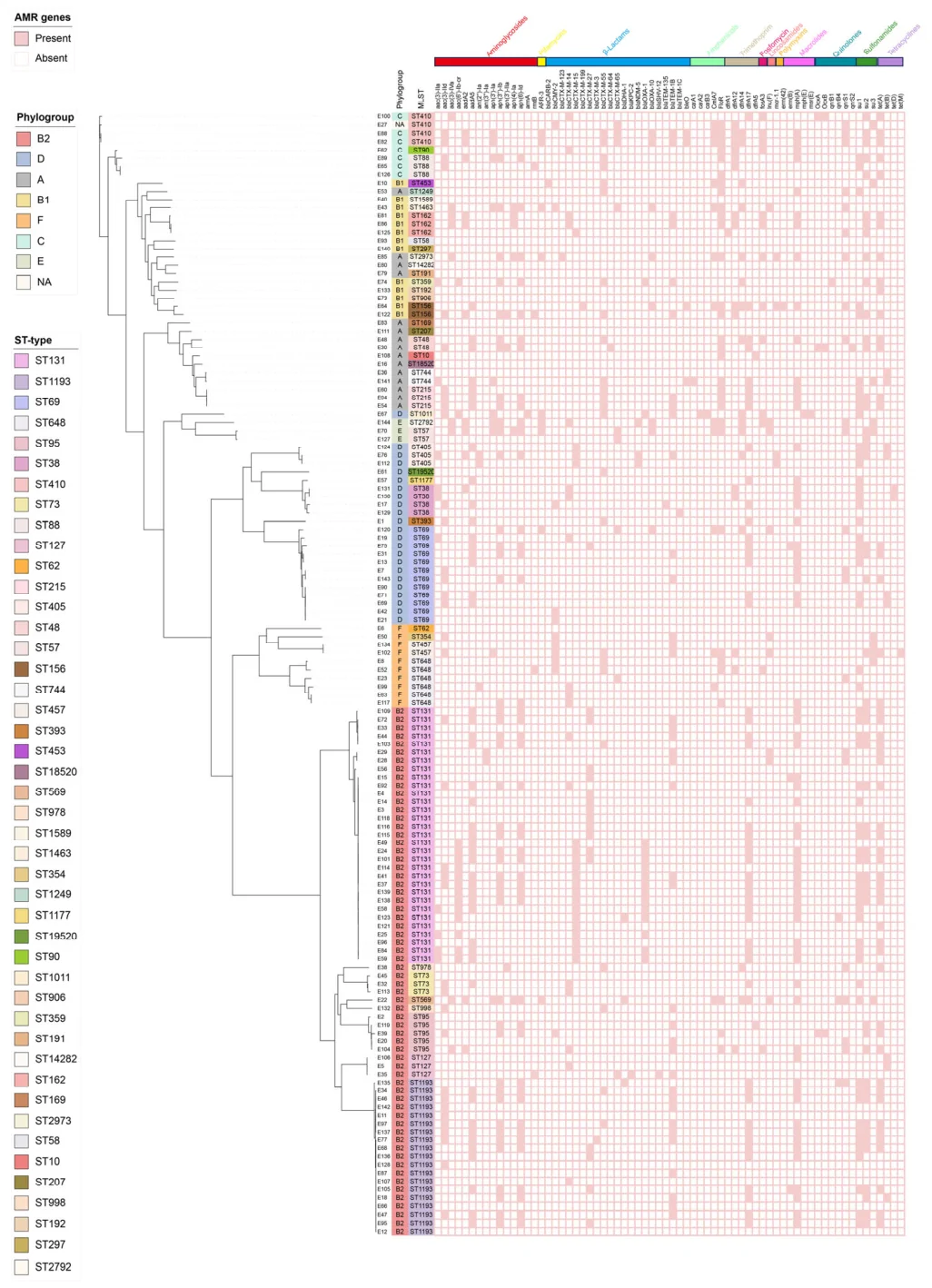
Core-genome phylogenetic tree with a corresponding ARG heatmap for 136 *E. coli* strains. The ARG genotype is colour-coded in the heatmap, with green indicating presence and pink indicating absence. Each strain is labeled with its phylogenetic lineage and sequence type. The gene names and their predicted antimicrobial resistance classes are marked above the diagram. The detection rates of ARGs differed by antibiotic classes in the 136 strains: β-lactamases (81%), aminoglycosides (67%), sulfonamides (62%), trimethoprim (57%), tetracyclines (46%), macrolides (49%), amphenicols (24%), quinolones (20%), fosfomycin (10%), rifamycin (7%), lincosamides (7%), and polymyxins (3%).

**Figure 5:**
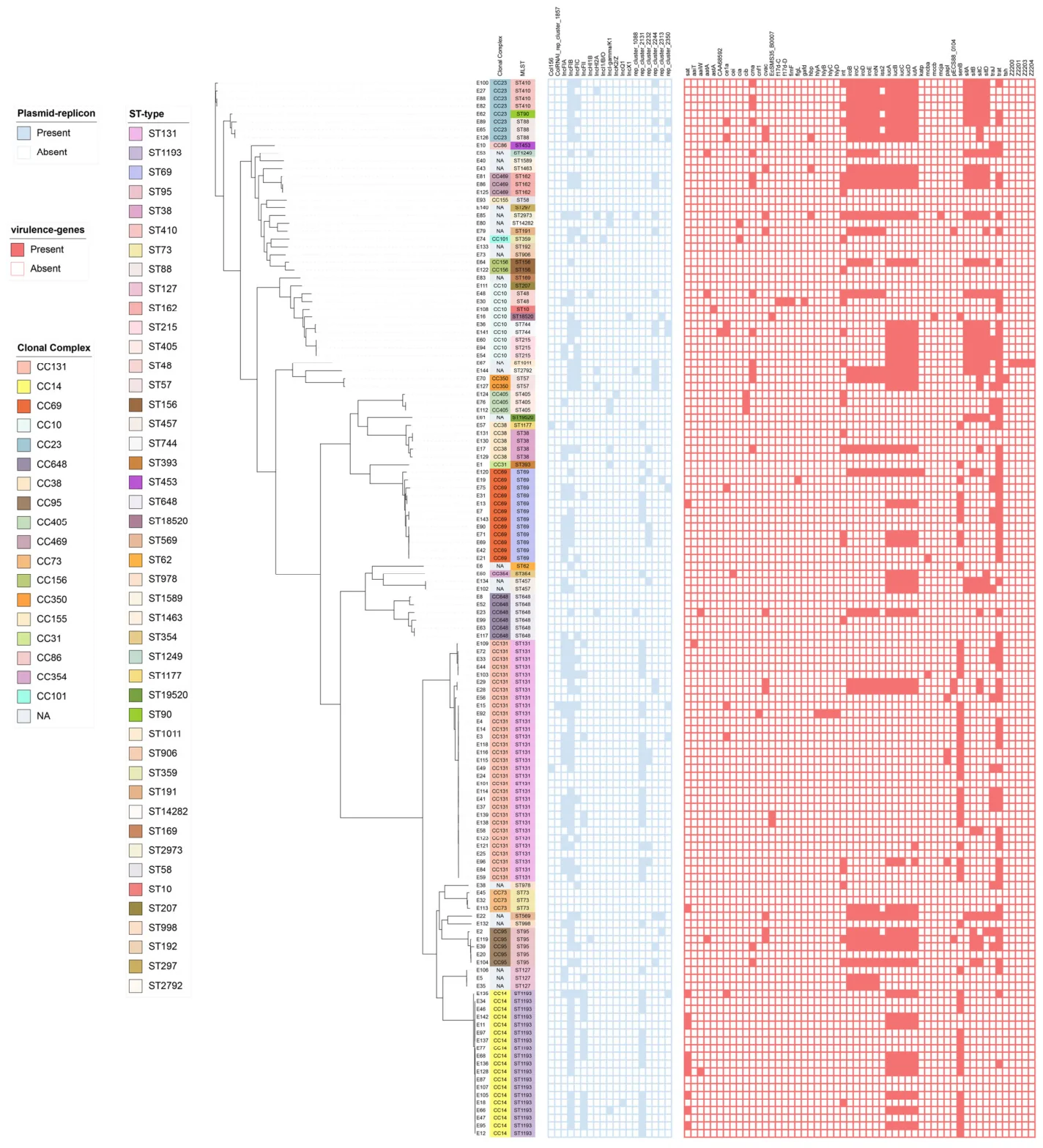
Colocalization of plasmid replicons and plasmid-borne virulence genes. A heatmap of plasmid replicon types and associated virulence genes is shown alongside the phylogenetic tree of the strains. Colocalization between specific replicons and virulence genes is colour-coded as indicated, with blue representing the presence of plasmid replicons and red representing the presence of plasmid-borne virulence genes. Each strain is labelled with phylogenetic lineage, sequence type, and serotype.

**Figure 6:**
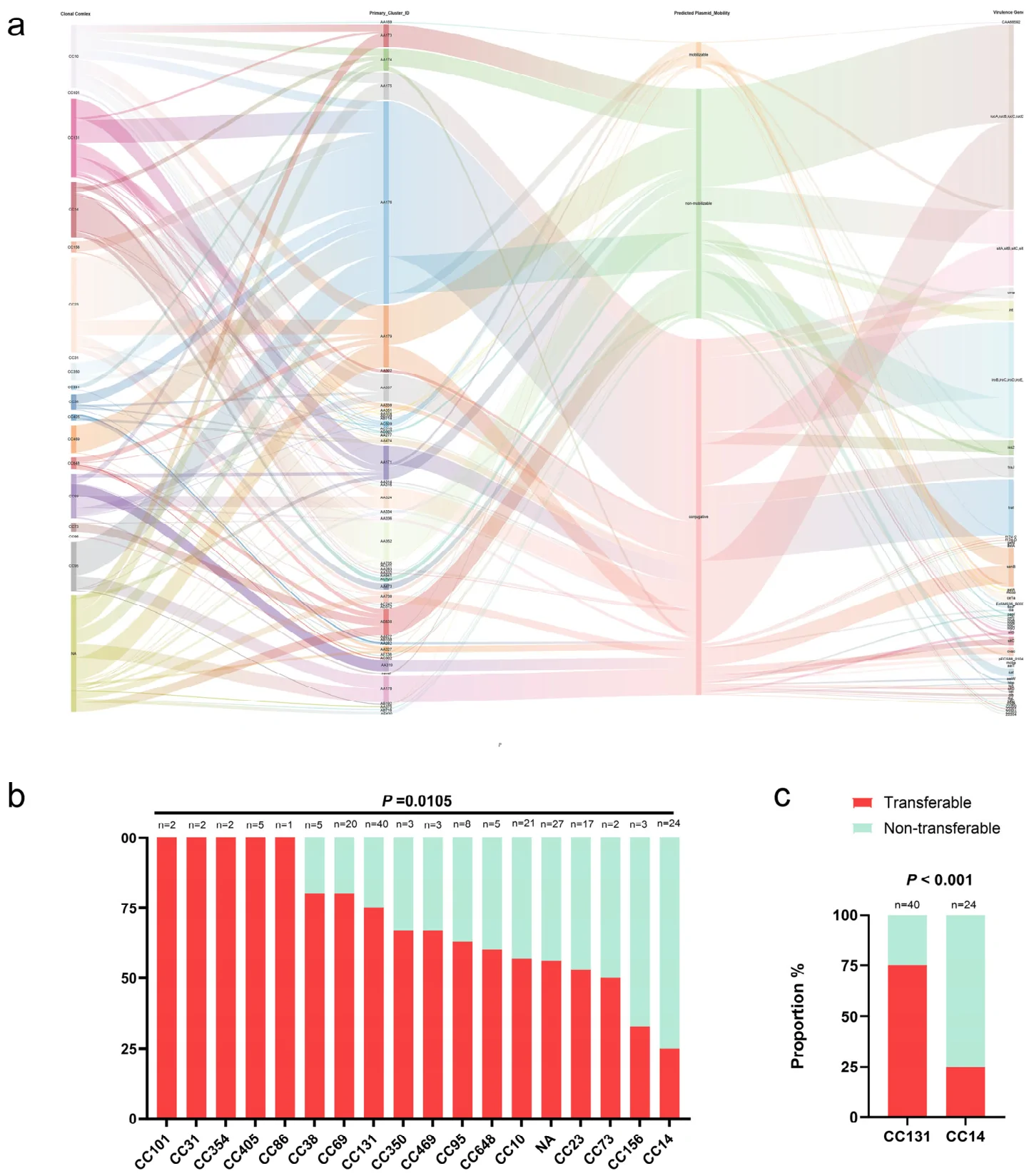
Distribution and predicted mobility of plasmid-associated virulence genes across clonal complexes. (**a**) Sankey diagram depicting the distribution and association of plasmid-associated virulence genes across clonal complexes and plasmid clusters. Predicted mobility components were divided into conjugative, mobilizable, or non-mobilizable. CC14 showed a significant correlation with AA352 plasmid clusters. A large proportion of plasmids in the AA352 cluster (*n* = 13/19, 68.4%) were found in CC14. Interestingly, all constituent AA352 plasmid clusters in CC14 carried the IncFIB replicon and were predicted to be non-transferable. The AA176 plasmid cluster carried the largest number of virulence genes, and 81% (*n* = 17/21) of plasmids in the AA176 cluster were predicted to possess mobility potential (conjugative or mobilizable), whereas all AA352 plasmids were predicted to be non-mobilizable. (**b**) The distribution of plasmid mobility differed significantly across clonal complexes (Fisher’s exact test with Monte Carlo simulation, *p* = 0.0105). Clonal complexes are ordered by the proportion of predicted mobile plasmids, and the number of plasmids per clonal complex is indicated above each bar. (**c**) Comparison of predicted plasmid mobility between CC131 and CC14. CC131 harbored a significantly higher proportion of predicted mobile plasmids than CC14 (30/40, 75.0% vs. 6/24, 25.0%; Fisher’s exact test, *p* < 0.001). The shared legend applies to panels B and C. No conjugation experiments were performed. Plasmid mobility was predicted using MOB-typer. A two-sided *p* value < 0.05 was considered statistically significant.

**Table 1:** Clinical characteristics of 136 *E. coli* blood-stream infection cases.

Characteristics	Number (%) ^&^
Demographics	
Age (years), median (IQR)	69 (60.0–78.6)
Age ≥ 60 years	105 (77.2)
Gender, male	72 (52.9)
Class, HA	86 (63.2)
Origin, primary	54 (39.7)
Origin, secondary	82 (60.3)
Source of BSIs	
GU tract infection	36 (43.9)
Skin and soft tissue infection	20 (24.4)
Non-biliary intra-abdominal infection	11 (13.4)
Respiratory tract infections	8 (9.8)
Biliary intra-abdominal infection	7 (8.5)
Comorbidities	
Endocrine, nutritional, and metabolic diseases	44 (32.4)
Diabetes mellitus	33 (24.3)
Disease of the circulatory system	71 (52.2)
Hypertension	60 (44.1)
Disease of the respiratory system	22 (16.2)
Pulmonary infection	18 (13.2)
Malignancy	29 (21.3)
Genitourinary disease	42 (30.9)
Recurrent urinary tract infection	20 (14.7)
Biliary tract diseases	18 (13.2)
Skin and soft tissue infection	14 (10.3)
Neurological-disease	12 (8.8)
Bone-fracture	19 (14)
Invasive procedures/devices	
Surgery	51 (37.5)
Paracentesis	10 (7.4)
AC	61 (44.9)
CVC	21 (15.4)
Endotracheal-tube	21 (15.4)
Invasive ventilation	17 (12.5)
Urinary catheter	58 (42.6)
Gastric tube	17 (12.5)
Drainage tube	24 (17.6)
Drug usage	
Corticosteroids	28 (20.6)
Antibiotics	118 (86.8)

Abbreviations: BSIs, bloodstream infections; IQR, interquartile range; HA, hospital-acquired; AC, arterial catheter; CVC, central venous catheter. ^&^ Number of cases with the characteristics, unless stated otherwise.

**Table 2:** Drug resistance phenotype and genetic characteristics of drug resistance genes in 136 *E. coli* strains.

Antibiotics Categories	Antimicrobial Agent	R (%)	Genetic Resistance Determinant
β-Lactam Antibiotics	Ampicillin	85.3	*blaCARB-2, blaCMY-2, blaCTX-M-123, blaCTX-M-14, blaCTX-M-15, blaCTX-M-199, blaCTX-M-27, blaCTX-M-3, blaCTX-M-55, blaCTX-M-64, blaCTX-M-65, blaDHA-1, blaKPC-2, blaNDM-5, blaOXA-1, blaOXA-10, blaSHV-12, blaTEM-135, blaTEM-1B, blaTEM-1C*
	Cefazolin	71.3	
	Cefuroxime	67.6	
	Ceftriaxone	66.9	
	Cefepime	40.4	
	Aztreonam	37.5	
	Ceftazidime	27.2	
	Ampicillin/Sulbactam	26.5	
	Amoxicillin/Clavulanate	20.6	
	Cefoxitin	12.5	
	Piperacillin/Tazobactam	11	
	Imipenem	2.9	
	Ertapenem	2.9	
	Meropenem	2.2	
Aminoglycosides	Gentamicin	42.6	aac(3)-IIa, aac(3)-IId, aac(3)-IVa, aac(6′)-Ib-cr *, aadA2, aadA5, ant(2″)-Ia, ant(3″)-Ia, aph(3″)-Ib, aph(3′)-Ia, aph(3′)-IIa, aph(4)-Ia, aph(6)-Id, rmtB, armA
	Tobramycin	28.7	
	Amikacin	3.7	
Co-trimoxazole	Compound Sulfamethoxazole	58.8	*sul1, sul2, sul3*, *dfrA1, dfrA12, dfrA14, dfrA17, dfrA5*
Quinolones	Ciprofloxacin	63.2	*qnrB1*, *qnrB4*, *qnrS1*, *qnrS2*, *OqxA*, *OqxB*
	Levofloxacin	62.5	
Tetracyclines	Tetracycline	61	*tet(A), tet(B), tet(D), tet(M)*
	Minocycline	2.2	
Others	Not included in the AST panel	NA	*catA1*, *catA2*, *catB3*, *CmlA7, FloR* *erm(42)*, *erm(B)*, *mph(A)*, *mph(E)*, *msr(E)*, *fosA3*, *lnu(F)*, *bleO*, *mcr-1.1*, *ARR-3*

Notes: R, resistant. * The *aac (6′)-Ib-cr* is a bifunctional resistance gene that confers resistance to both aminoglycosides and quinolones. In our study, we classified it under aminoglycosides based on its core function.

**Table 3:** The concordance between the resistance phenotype and the predicted genotype of 136 *E. coli* isolates.

Antibiotic Categories	Antibiotics	Phenotype: Resistance		Phenotype: Susceptible		Cohen’s Kappa (κ) (95% CI)	Agreement
		Genotype: Resistant	Genotype: Susceptible	Genotype: Resistant	Genotype: Susceptible		
β-Lactam Antibiotics	AMP	109	7	1	19	0.791 (0.654–0.928)	Substantial
	KZ	92	5	3	36	0.858 (0.764–0.952)	Almost perfect
	CXM	86	6	9	35	0.743 (0.621–0.865)	Substantial
	CRO	83	8	4	41	0.805 (0.699–0.911)	Almost perfect
	FEP	53	2	34	47	0.497 (0.372–0.622)	Moderate
	ATM	49	2	38	47	0.450 (0.325–0.575)	Moderate
	CAZ	37	0	58	41	0.278 (0.180–0.376)	Fair
	SAM	24	12	8	92	0.608 (0.453–0.763)	Substantial
	AMC	24	4	8	100	0.744 (0.607–0.881)	Substantial
	FOX	14	3	4	115	0.770 (0.607–0.933)	Substantial
	TZP	12	3	26	95	0.350 (0.179–0.521)	Fair
	IPM	4	0	0	132	1.000 (1.000–1.000)	Almost perfect
	ETP	4	0	0	132	1.000 (1.000–1.000)	Almost perfect
	MEM	3	0	1	132	0.853 (0.571–1.000)	Almost perfect
Aminoglycosides	GEN	53	5	6	72	0.835 (0.741–0.929)	Almost perfect
	TOB	34	5	31	66	0.460 (0.321–0.599)	Moderate
	AK	5	0	14	117	0.381 (0.140–0.622)	Fair
Co-trimoxazole	SXT	71	9	2	54	0.836 (0.744–0.928)	Almost perfect
Quinolones	CIP	84	2	2	48	0.937 (0.876–0.998)	Almost perfect
	LEV	83	2	3	48	0.921 (0.852–0.990)	Almost perfect
Tetracyclines	TET	52	31	11	42	0.392 (0.245–0.539)	Fair
	MH	1	2	9	124	0.124 (−0.141–0.389)	Slight

The concordance between the resistance phenotype obtained by AST and the predicted genotype was assessed using Cohen’s kappa coefficient. Landis and Koch benchmarks for kappa: 0.81–1.00, almost perfect; 0.61–0.80, substantial; 0.41–0.60, moderate; 0.21–0.40, fair; 0.01–0.20, slight; ≤0, no agreement.

## Data Availability

All raw sequencing data generated in this study are publicly available in the NCBI database (BioProject: PRJNA1390407).

## Supplementary Materials

The following supporting information can be downloaded at: https://www.mdpi.com/article/10.3390/antibiotics15100940/s1, Figure S1: Phenotypic detection of carbapenemase production; Figure S2: Genotypic landscape of quinolone resistance in isolates; Table S1: Individual-level clinical data for all 136 patients and st-types of strains; Table S2: Sequencing and assembly quality information of 136 strains.
