# Supplementary material for: Genomic Insight into Escherichia coli Bloodstream Infections at a Tertiary Hospital in Shanghai: Predominance, Genetic Diversity, and Plasmid-Associated Virulence of CC131 and CC14
Source: Antibiotics (Basel). 2026 Sep 22;15(10):940. doi: 10.3390/antibiotics15100940 (PMC13700793; doi:10.3390/antibiotics15100940)
Supplement: Supplementary file 1 [file antibiotics-15-00940-s001.zip › Supplementary Figure.pdf]

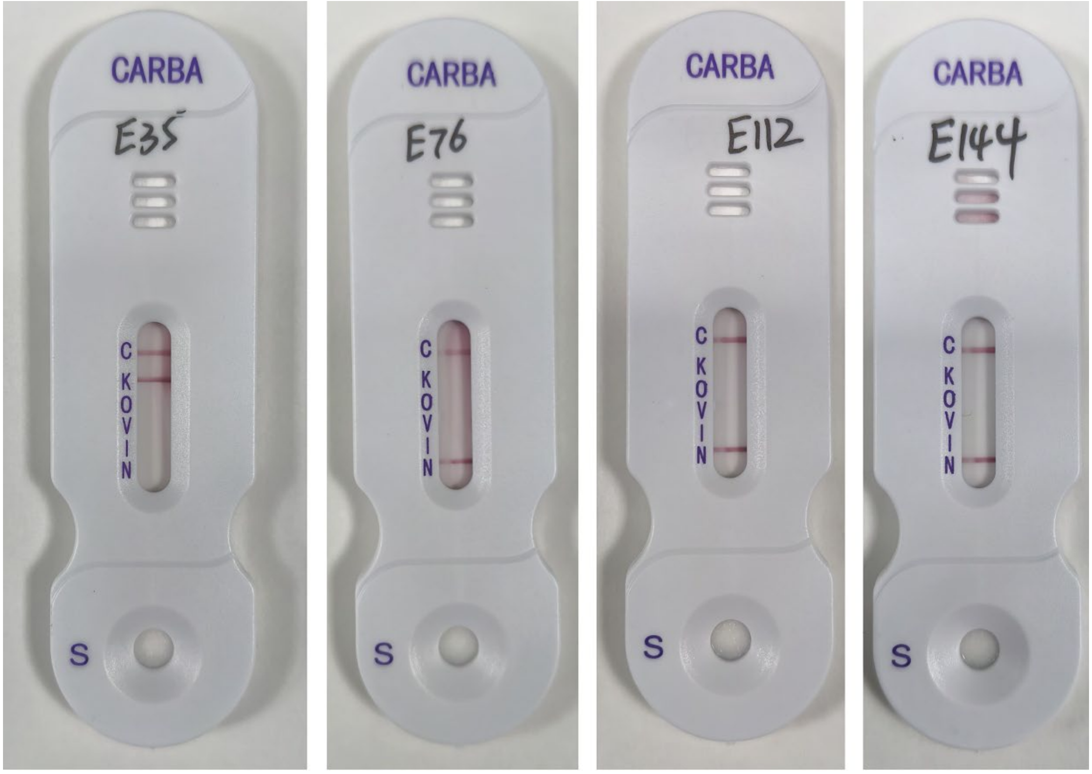

a b c d

Supplementary Figure S1.Phenotypic detection of carbapenemase production.
All 4 carbapenemase gene-carrying isolates tested positive for carbapenemase production

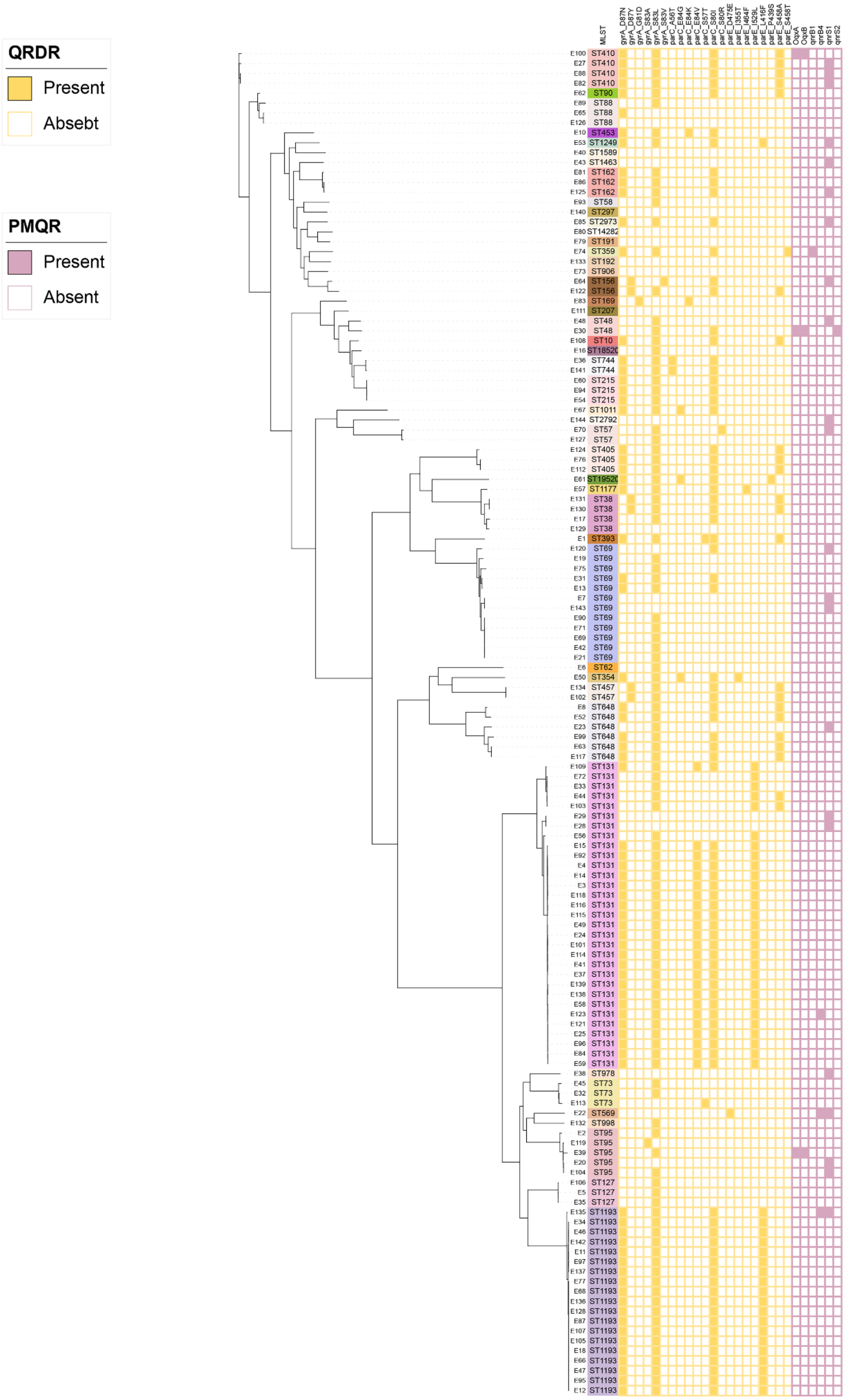

QRDR
Present
Absebt
PMQR
Present
Absent
MLST
gyrA_D87N
gyrA_D87Y
gyrA_G81D
gyrA_S83A
gyrA_S83L
gyrA_S83V
parC_A56T
parC_E84G
parC_E84K
parC_E84V
parC_S57T
parC_S80I
parC_S80R
parE_D475E
parE_I355T
parE_I464F
parE_I529L
parE_L416F
parE_P439S
parE_S458A
parE_S458T
OqxA
OqxB
qnrB1
qnrB4
qnrS1
qnrS2

Supplementary Figure S2: Genotypic landscape of quinolone resistance in isolates. The heatmap illustrates the presence of Quinolone Resistance-Determining Region (QRDR) mutations and Plasmid-Mediated Quinolone Resistance (PMQR) genes.
